# Preparation of a novel nitrogen-containing graphitic mesoporous carbon for the removal of acid red 88

**DOI:** 10.1038/s41598-020-57823-z

**Published:** 2020-01-28

**Authors:** Qiying Zhou, Wenhua Chen, Xia Jiang, Hongying Liu, Shenggui Ma, Bangda Wang

**Affiliations:** 1 College of Architecture and Environment, Sichuan University, Chengdu, 610065 China; 2 National Engineering Research Centre for Flue Gas Desulfurization, Chengdu, 610065 China

**Keywords:** Environmental sciences, Materials science

## Abstract

A novel nitrogen-containing graphitic mesoporous carbon was prepared through MnO-templated method using polyacrylonitrile (PAN) as both carbon and nitrogen sources, and MnCO_3_ as both template and catalyst precursors. The effects of preparation conditions on the physicochemical properties of obtained samples were systematically investigated. The results showed that as the decrease of the weight ratios of PAN and MnO (2:1–1:4), the increase of carbonization temperature (700–900 °C) and pre-oxidation temperature (180–200 °C), the samples had higher specific surface area, mesopores volume and ratios, up to 507 m^2^/g, 0.824 cm^3^/g and 96.83%, respectively. Moreover, the prepared samples presented relatively high graphitic degree and nitrogen contents (~2.21%). The adsorption capacity for acid red 88 (AR88) was as high as 309 mg/g, which were dramatically affected by the mesoporous properties and C- and N-containing groups on the surface of prepared carbon. The rich graphic carbon and pyridine-N in mesoporous carbon generated π-π dispersion and electrostatic interaction with AR88, respectively, which jointly were responsible for the adsorption process. The results of the isotherm and kinetic studies indicated that the AR88 adsorption on mesoporous carbon could be well depicted using Langmuir model and pseudo-2^nd^-order model.

## Introduction

Recently, the demand for dyes is increasing, with the fast development of industries, including textile, printing, paper making, cosmetic, etc. The dye wastewater has gradually become one of the vital pollutants, which seriously affects both human beings’ health and environment. Thus, it is significantly important for the removal of dye pollutants from wastewater before discharging into environment. However, it is a great challenge to treat dye wastewater, because of its high resistance to photo-degradation, oxidation and bio-degradation^1^. It was reported that the adsorption by mesoporous carbon (i.e. pores size from 2 to 50 nm) is a desirable method for the removal of dye pollutants with large molecules diameters, with many advantages such as easy operation, high efficiency, and the recycle of dye^2–4^. In comparison with the microporous adsorbent, the mesoporous carbon possessed higher adsorption capacity for the large dye molecules^5–7^. The adsorption rate could also be significantly enhanced by introducing the mesoporous structure into the carbon materials^8^.

Normally, various hard templates including zeolites, mesoporous silica are used to prepare the mesoporous carbon by removing these ordered structural frameworks. This strategy has been given extensive attention in the literature due to the high stability of templates and precise control for porous structure^9^. However, when removing these hard templates, some strong corrosive agents like hydrofluoric acid and sodium hydroxide are used, which will always cause unavoidable corrosion, and the templates are obliged to be wasted^10^. In this way, the high preparation cost of the template method seriously hinders its application in practical industry. Therefore, developing a clean and efficient template is significant for the preparation of mesoporous carbon. MnO, as an alternative template, shows high thermal stability and the tunable pore size can be easily given by decomposing its precursors. Then the pores are attained by isolating the generated nano-MnO using the diluted low-corrosive acid solution, like HCl and H_2_SO_4_. More importantly, the MnO templates can be cyclically utilized from the pickling solution. Thus, this strategy simultaneously enhances the operation safety and lowers the preparation cost.

On the other hand, the chemically inert and poorly hydrophilic surface of mesoporous carbon severely limited its application as the efficient absorbent for dye pollutants. Thus, it is necessary to develop some useful strategies to realize the surface modification of mesoporous carbon. Recently, massive methods^11^ have been applied to improve the surface properties of the mesoporous carbon, among which, nitrogen doping is considered as an effective one and has attracted increasing attention. Previous studies showed that both the adsorption capacity and the rate of the anionic dye by carbon materials could be enhanced significantly when the nitrogen-containing groups were doped on the surface of carbon^12,13^. There are two common approaches to introduce nitrogen into carbon, i.e. NH_3_ gas post-modification and *in situ* doping. However, the post-modification could increase the complexity and the cost of the preparation process, together with high corrosivity during the operation process, thus severely inhibiting its wide application. In contrast with the post-modification, the *in situ* procedure can ensure the homogeneous doping of nitrogen throughout the entire mesoporous carbon material^14^. It could be a suitable method for the preparation of N-doped carbon material by using the carbon materials with a high content of nitrogen^15^. Polyacrylonitrile (PAN) is a kind of polymer material with a high content of C≡N functional group, which presents great potential as both carbon and nitrogen precursors for the preparation of N-doped mesoporous carbon^16^. Liu *et al*.^17^ fabricated N-doped ordered porous carbon by the nano-casting process using PAN as carbon and nitrogen precursors, with nitrogen content up to 6.88%. In addition, it was reported that manganese compounds presented an excellent catalyst for the graphitization of carbon at around 1000 °C, i.e. manganese oxides^18,19^. Such graphitic structure could promote the adsorption of dye molecules with aromatic ring via π-π dispersion interaction^20,21^. However, few studies have been conducted on using manganese salts as both template and catalyst precursors for the preparation of graphitic mesoporous carbon.

Thus, in this study, a novel nitrogen-containing graphitic mesoporous carbon was prepared by MnO-template method using PAN as both carbon and nitrogen sources, together with commercial MnCO_3_ as both template and catalyst precursors. The effects of MnCO_3_ on the carbonization and graphitization of PAN molecules was investigated. The influences of preparation conditions on the physicochemical properties and acid red 88 (AR88) adsorption process of prepared carbon were discussed, including weight ratios of PAN and MnO, carbonization temperature and pre-oxidation temperature. Finally, the isotherm and kinetics of the adsorption process were provided.

## Results

### Characterization of mesoporous carbon

#### TG analysis

**Figure 1:**
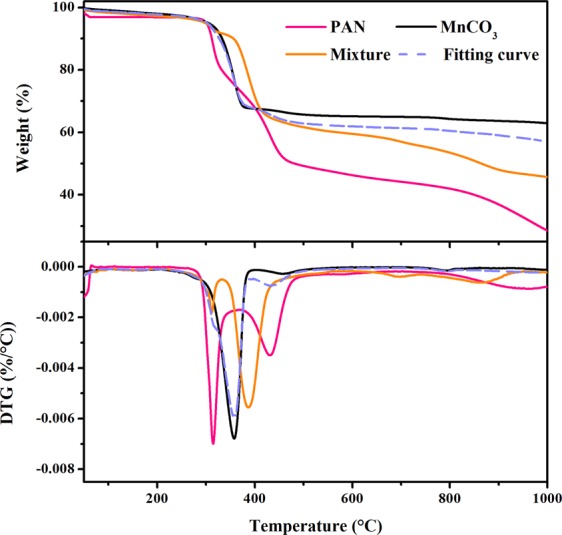
TG and DTG curves of PAN, MnCO_3_, their mixture with weight ratio of 1:3 and the related fitting curve.

Figure 1 presents the TG and DTG curves of PAN, MnCO_3_ and their mixture (weight ratio = 1:3) in N_2_ atmosphere. The pyrolysis of PAN could be divided into four stages. The first stage was from room temperature to 175 °C with about 4% of weight loss, which could be ascribed to the evaporation of H_2_O and acrylonitrile monomer remained on the surface of PAN. The second weight-loss stage happened from 175 °C to 350 °C, with about 31% of weight loss and the DTG peak located at 315 °C. In this stage, the -C≡N was transformed to -C=N with the escape of HCN and the generation of C=C^22^. The third weight-loss stage from 350 to 500 °C was corresponded to the hemolytic cleavage, cyclization and aromatization of PAN, with the release of HCN, NH_3_ and some N-containing light alkenes, resulting in the loss of H and N atoms and residue of C-rich pyrolysis solid^23^. After 500 °C, the fourth stage occurred, with about 30% of weight loss, especially over 800 °C, which could be ascribed to the elimination of non-carbon atoms and realignment of the carbon structure. However, definite graphitization is not possible for the carbonized PAN without pre-oxidation and catalyst at this temperature range. As clearly seen in Fig. 1, only one weight-loss stage could be observed during the pyrolysis of MnCO_3_ within 1000 °C, from 300 to 390 °C with about 37% of weight loss, and the DTG peak was located at 358 °C. Based on the ratio of weight loss, it could be assumed that the decomposition products of MnCO_3_ were MnO and CO_2_. In addition, no further weight loss occurred after 390 °C, which indicates that the produced MnO kept stable within 1000 °C.

To analyze the effect of MnCO_3_ on the decomposition of PAN, the fitting TG and DTG curves were calculated from the curves of MnCO_3_ and PAN with the weight ratio of 1:3. Before 320 °C, the TG curves of the fitting and the mixture were coincided, indicating that no interactions occurred between PAN and MnCO_3_ within this temperature range. When the temperature got higher, the two curves started to diverge. From 320 and 400 °C, the weight loss of the mixture was delayed, compared with the fitting curve, which might be ascribed to the inhibition of PAN pyrolysis or MnCO_3_ decomposition. However, from 480 to 1000 °C, the mixture curve presented much more weight loss (about 11%) than the fitting one, indicating that the existence of MnO could accelerate the decomposition of PAN at a higher temperature.

The TG and DTG curves of the mixture after pre-oxidation (200 °C for 1 h) are illustrated in Fig. S1. It can be seen that the pre-oxidized mixture presented similar DTG peaks to one without pre-oxidation, while their weight loss curves were slightly different. Even though the reaction degree was very low in this period, they are vital for the cyclization of PAN molecules in the following carbonization and could help reduce the weight loss^24^. Zhao *et al*.^25^ reported that during the pre-oxidization process at around 200 °C, the PAN was converted to an infusible stable ladder polymer by the movement of C≡N in acrylonitrile units into C=N bonds and the generation of C=O groups in comonomer units.

#### TEM and XRD analysis

**Figure 2:**
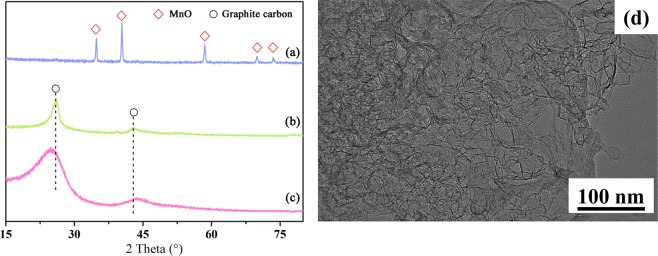
XRD patterns of carbonized samples (the mixture with the weight ratio of PAN and MnO at 1:3 before (**a**) and after acid washing (**b**), carbonized PAN (**c**)); high-resolution TEM image of carbonized samples after acid washing.

The XRD patterns of the carbonized sample at 900 °C before and after acid washing are shown in Fig. 2. It can be seen that the peaks at 2θ = 34.9°, 40.55°, 58.72°, 70.18° and 73.79° could be observed for the carbonized sample before acid washing, which was ascribed to MnO. This indicates that MnO was the only crystalline substance existing in the carbonized sample. Combined with the TG analysis (Fig. 1), it could be confirmed that MnO kept stable during the carbonization process.

As shown in Fig. 2, after acid washing of the carbonized sample, the diffraction peaks of Mn compounds disappeared, while only the peaks ascribed to graphite carbon could be detected. This reveals that the MnO was completely removed and the carbonized sample presented a clearly graphitized structure. The XRD pattern of the carbonized pristine PAN with the same conditions of mixture sample was also given, whose peaks belonging to graphitized carbon were evidently broader than those of the sample with the addition of MnCO_3_. This indicates that after the addition of MnCO_3_, the crystallization degree of the mixture sample was clearly higher. Furthermore, the (002) diffraction plane of the samples with and without MnCO_3_ addition appeared at 2θ = 26.08 and 24.96°, respectively, with the corresponding d_002_ = 3.41 Å and 3.57 Å. It was reported that the perfect hexagonal graphite has a d_002_ value of 3.35 Å, suggesting that the mixture sample has a better-graphitized structure. As shown in TG analysis (Fig. 1), the existence of MnO could promote the decomposition of PAN during 480 to 1000 °C, which could result in the difference on carbon structure between the samples with and without MnCO_3_. This confirmed the catalysis effect of MnO on the graphitization of PAN.

Oya and Marsh^26^ proposed four types of effects of catalytic graphitization, including G-effect, Ts-effect, A-effect and Tn-effect. As shown in Fig. 2, the peaks belonging to graphite carbon for the mixture sample were symmetrical and well-proportioned, suggesting that the effects of MnO catalytic graphitization on PAN seemed to be due to the A-effect. It was reported that the A-effect was caused by the catalyst with ultra-fine diameters, like the metallic vapour, which could result in a more homogenous effect of catalysts for carbon precursors. However, in this study, no Mn species could be gasified within this temperature range (below 900 °C), suggesting that there might be some other effects existed. The porous structure created in carbons was further studied using the transmission electron microscope (TEM) observation and the related images were shown in Fig. 2d. It can be clearly seen from Fig. 2d, the prepared carbon after acid washing shows well-developed porous structure with abundant mesopores.

#### Textural properties

The N_2_ adsorption-desorption isotherms of the prepared carbon are given in Fig. S2. All curves presented a low N_2_ adsorption capacity at a lower relative pressure (below 0.01) and a remarkable uptake when the relative pressure was higher than 0.4. An evident hysteresis loop could be observed, indicating that all of the prepared carbon could be classified as mesoporous materials.

The porous properties of prepared carbons calculated from the isotherms were summarized in Table 1. It can be seen that their S_BET_ increased from 164 to 507 m^2^/g with the decrease of the weight ratios of PAN and template from 2:1 to 1:4, while subsequently declined to 455 m^2^/g when the ratio was 1:5. Their V_tot_ and V_meso_ also increased from 0.399 and 0.381 cm^3^/g to 0.881 and 0.854 cm^3^/g, respectively, with the weight ratios ranging from 2:1 to 1:3, while dropped to 0.745 and 0.722 cm^3^/g when the ratio was 1:5. This indicates that the template was filled with the inner parts of carbon when the ratio was about 1:3. The V_meso_/V_tot_ ratios of all samples were high, above 95%, while their V_mic_ was evidently low (i.e. 0.018–0.027 cm^3^/g) and roughly invariant with the change of template contents. This suggests that the micropores are created caused by the decomposition process of the carbon precursor (i.e. PAN), while the mesopores were from the templates.

**Table 1:** Textural properties of prepared samples.

Preparation condition	Value	S_BET_ (m²/g)	V_tot_ (m^3^/g)	V_mic_ (cm^3^/g)	V_meso_ (cm^3^/g)	V_meso_/V_tot_ (%)
Weight ratio (PAN:MnO)	2:1	164	0.399	0.018	0.381	95.49
	1:1	282	0.686	0.026	0.660	96.21
	1:2	411	0.705	0.021	0.684	97.02
	1:3	489	0.881	0.027	0.854	96.94
	1:4	507	0.851	0.027	0.824	96.83
	1:5	455	0.745	0.023	0.722	96.91
Carbonization temperature (°C)	700	45	0.040	0.016	0.024	60.00
	800	371	0.464	0.024	0.440	94.83
	900	489	0.881	0.027	0.854	96.94
	950	472	0.859	0.026	0.833	96.97
Pre-oxidation temperature (°C)	—	362	0.604	0.028	0.576	95.36
	180	439	0.749	0.022	0.727	97.06
	200	489	0.881	0.027	0.854	96.94
	240	337	0.449	0.026	0.423	94.21
After adsorption	—	127	0.369	0.000	0.369	100

**Figure 3:**
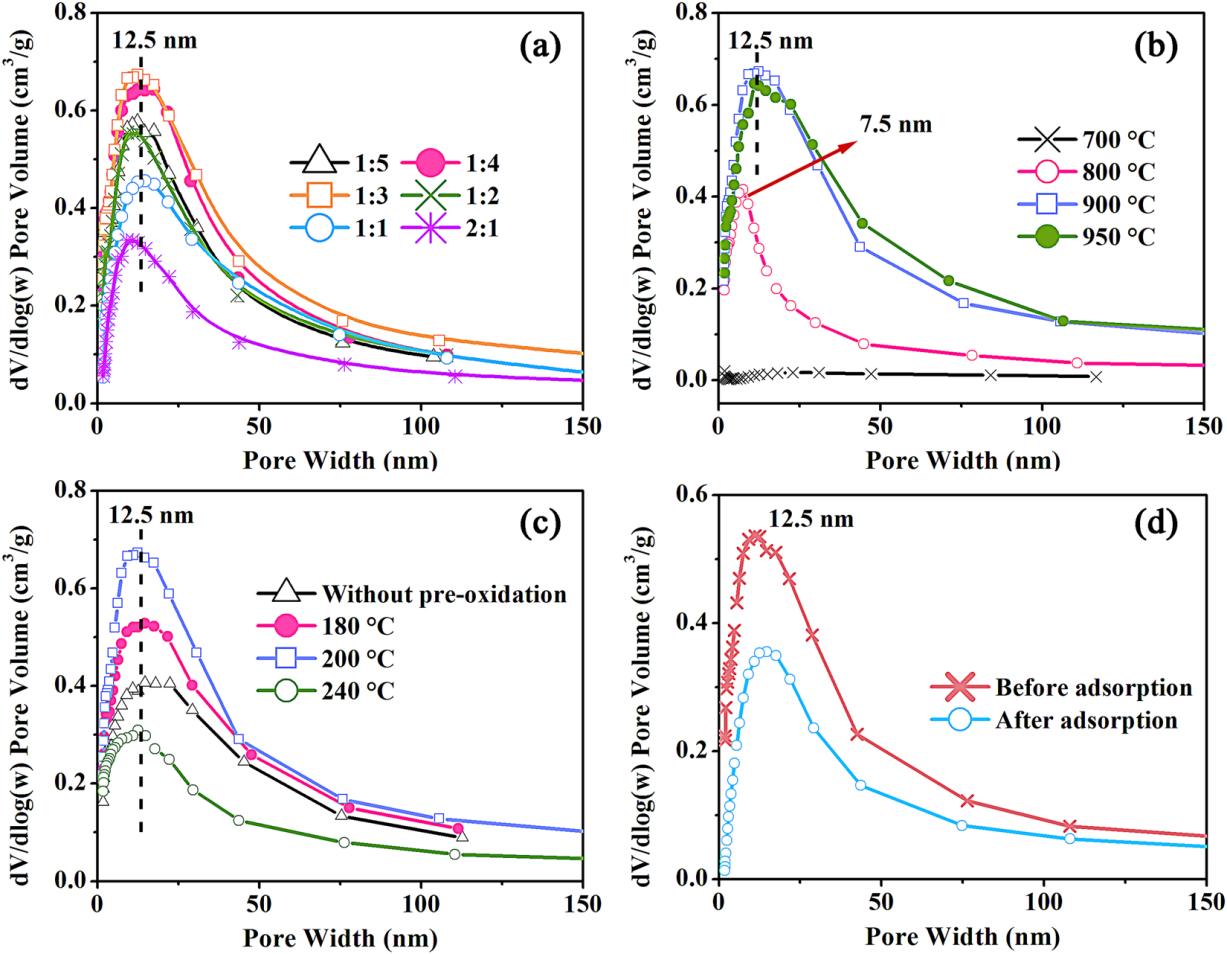
Mesopore size distribution of the samples prepared from different conditions (Legends represented the weight ratio of PAN and MnO (**a**), carbonization temperature (**b**), and pre-oxidation temperature (**c**), before and after adsorption of the mesopore carbon with the weight ratio of 1:3, carbonized at 900 °C and pre-oxidized at 200 °C (**d**)).

Their mesopores size distributions are plotted in Fig. 3(a). The prepared carbon exhibited a relatively narrow pore size distribution with a peak at around 12.5 nm. Furthermore, with decreasing the weight ratio of PAN and MnO from 2:1 to 1:3, the height of the peaks gradually increased first and then dropped a little when the weight ratios were lower than 1:4. This also indicates that the mesopores were generated from the removal of the template and the weight ratio of PAN and template was the key factor affecting the mesopore contents.

The textural properties of mesoporous carbon prepared at different temperature are also tabulated in Table 1. The S_BET_, V_tot_ and V_meso_ of the sample carbonized at 700 °C were only 45 m^2^/g, 0.040 cm^3^/g and 0.024 cm^3^/g, respectively, suggesting that little pores could be produced when carbonization temperature was 700 °C (Fig. S2(b)). The porous structure of the samples with carbonization temperature over 800 °C turned to be evidently richer, with S_BET_, V_tot_ and V_meso_ at 489 m^2^/g, 0.881 cm^3^/g and 0.854 cm^3^/g, respectively, for the sample at 900 °C. With a further increase of carbonization temperature at 950 °C, the porous structure did not change significantly. For V_mic_, the sample carbonized at 700 °C exhibited the lower V_mic_ (0.016 cm^3^/g), while the V_mic_ increased slightly to 0.024–0.027 cm^3^/g for those carbonized over 800 °C, indicating that more micropores from the decomposition of PAN could be obtained at a higher temperature.

Figure 3(b) provides the variation of the mesopore size distribution of the samples prepared from different carbonization temperature. No peak could be observed for the sample carbonized at 700 °C, while an evident peak at 7.5 nm could be found for that at 800 °C. When the carbonization temperature reached 900 °C, a broader peak at around 12.5 nm could be detected, which might be attributed to the agglomeration of nano-MnO particles at a higher temperature. This reflects that carbonization temperature is also an essential factor determining the porous structure of prepared carbon. As shown in TG analysis (Fig. 1), the last weight-loss stage of the mixture sample took place at around 860 °C, revealing that the decomposition of PAN was not completed before this temperature, which results in the low V_mic_ for that carbonized at 700 °C. Such poor microporous structure might block the H^+^ entering into the inner parts of carbon and accordingly, MnO would not be removed by acid washing, causing a poor mesoporous structure (Table 1 and Fig. 3b).

The effect of pre-oxidation temperature on the porous structure of prepared samples was also discussed (Table 1). Compared with the sample without pre-oxidation, the samples pre-oxidized at 180 and 200 °C possessed a remarkably abundant porous structure, demonstrating that the pre-oxidation process could help the production of pores. Furthermore, with the increase of pre-oxidation temperature from 180 to 200 °C, their S_BET_, V_tot_ and V_meso_ increased and then declined when the temperature was higher than 200 °C. Their mesopore size distributions are presented in Fig. 3(c). All peaks were emerged at 12.5 nm, suggesting that the pre-oxidation temperature would not affect the particle size of generated nano-MnO. This might be ascribed to the higher decomposition temperature of MnCO_3_ (~ 358 °C) than the pre-oxidation temperature (<240 °C). However, their peak areas changed with the variation of pre-oxidation temperature, which might reflect that the pre-oxidation temperature could influence the carbon structure. As a result, this might affect the removal of MnO by acid washing. It was reported that the pre-oxidation mainly occurred in amorphous region at low temperature (<200 °C), while the crystalline area started to be oxidized at high temperature (>235 °C), resulting in the transformation of chain to trapezoid structure with higher thermal stability^27^. For the templated method, such stable carbon structure could lead to a coating of MnO templates, increasing the difficulty in the removal of templates. Thus, higher pre-oxidation temperature could lead to a poor porous structure of prepared samples.

### AR88 adsorption performance

**Figure 4:**
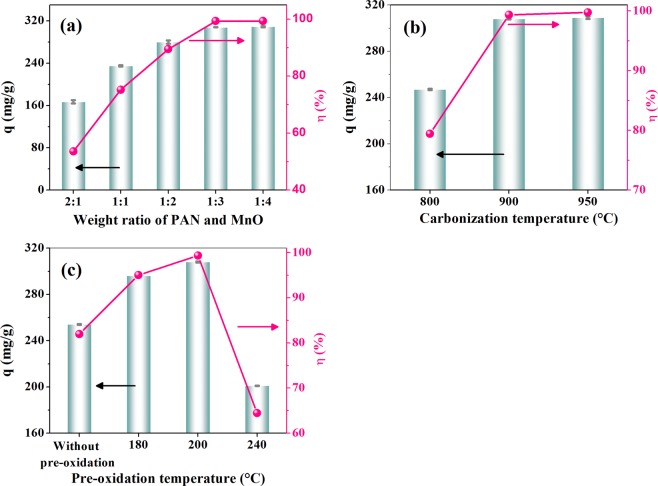
AR88 adsorption capacities of the samples prepared from different conditions (weight ratio of PAN and MnO (**a**), carbonization temperature (**b**), and pre-oxidation temperature (**c**). Error bars represent the standard deviation of triplicate experiments).

The mesoporous carbon prepared from different conditions was adopted as the adsorbents for the removal of AR88 and their results are presented in Fig. 4. With the decrease of PAN and MnO weight ratios from 2:1 to 1:4, the adsorption capacities and removal efficiencies of the dye increased steadily from 167 mg/g and 53.53% to 309 mg/g and 99.76%, respectively. When the carbonization temperature raised from 800 to 900 °C, the adsorption performance of the obtained sample was enhanced significantly. Furthermore, the samples with the pre-oxidation at 180 and 200 °C presented higher adsorption capacities of AR88 than that without pre-oxidation. With the increase of pre-oxidation temperature from 180 to 200 °C, the adsorption capacities increased slightly and then dropped evidently for the sample with the pre-oxidation temperature at 240 °C. The results show that high adsorption capacity and removal efficiency of the dye was obtained for the sample with the weight ratio of 1:3, the pre-oxidation temperature at 200 and carbonization temperature at 900 °C. Table 2 lists and compares the maximum adsorption capacity of AR88 on various adsorbents. The value of the maximum adsorption capacity determined from the Langmuir isotherm was 309 mg g^−1^, which was comparable to the published results in previous papers^28–35^. This result suggests that the PAN-mesoporous as adsorbent was suitable for the removal of AR88 from aqueous solution.

**Table 2:** Comparison of the AR88 adsorption performance on various adsorbents.

Adsorbent	q (mg g^−1^)	Ref.
PAN-mesoporous carbon	309	This study
Activated carbon	109	^28^
Multi-walled carbon nanotubes-Fe_3_C	54.4	^29^
ZnFe_2_O_4_	111.1	^30^
Azolla filiculoides	123.5	^31^
Anion exchange membrane	227.1	^32^
Graphene aerogel/CaCO_3_ composite	1234	^33^
Fe@graphite nanocomposite	41.8	^34^
Hollow mesoporous carbon nanospheres	555.6	^35^

### AR88 adsorption mechanisms

#### The relationship between adsorption capacity and surface chemistry

**Figure 5:**
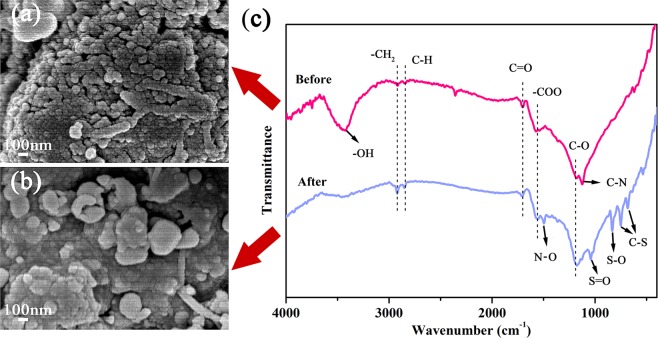
SEM images of prepared samples before (**a**) and after (**b**) adsorption of AR88; FTIR spectra (**c**) of the prepared sample (the weight ratio of 1:3, carbonized at 900 °C and pre-oxidized at 200 °C) before and after adsorption of AR88.

The elemental formation on the carbon was investigated by FTIR and XPS analysis. From the microstructure of the prepared sample (Fig. 5a), the mesoporous carbon presented the abundant and clearly visible porous structure. After the adsorption process, the surface of mesoporous carbon was covered and most tunnels were blocked, indicating the successful adsorption of AR88 molecules. As illustrated in FITR analysis (Fig. 5c), the bands emerged at 3700-3000, 2925, 2847, 1705, 1578 and 1182 cm^−1^ could be found for the prepared samples, which belongs to the stretching vibration of O-H from sugar, asymmetric stretching vibration of CH_2_, stretching vibration of C-H, stretching vibration of C=O, asymmetric stretching vibration of COO and stretching vibration of C-O. Moreover, the absorption band appeared at 1129 cm^−1^ could also be observed, indicating the existence of C-N that came from the transformation of C≡N during the carbonization process. After AR88 adsorption of the carbon, the intensity of C=O was weakened and the band of C-N disappeared, implying that they could take part in the adsorption process. Moreover, the bands located at 1041, 839, 751 and 685 cm^−1^ appeared, which were assigned to the stretching modes of SO_3_ in the sulfonate group, S-O and C-S, respectively^35^. This might come from the sulfonic group in AR88 molecules that was adsorbed on the carbon surface.

The elemental contents obtained from XPS spectra of carbon pre-oxidized at different temperature are listed in Table 3. With the increase of pre-oxidation temperature, the relative contents of oxygen gradually increased, demonstrating that more oxygen atoms were introduced to the surface of the carbon matrix. On the contrast, the N contents decreased with the growing of pre-oxidation temperature, especially for that at 240 °C. This indicated that more N species was released at a relatively higher pre-oxidation temperature.

**Table 3:** Elemental contents and q/S_BET_ of carbon pre-oxidized at different temperature.

Pre-oxidation temperature (°C)	Elemental analysis (%)			q/S_BET_
	C	O	N	
—				0.702
180	93.74	4.59	1.04	0.674
200	93.90	4.80	1.01	0.630
240	94.04	5.26	0.52	0.596

**Figure 6:**
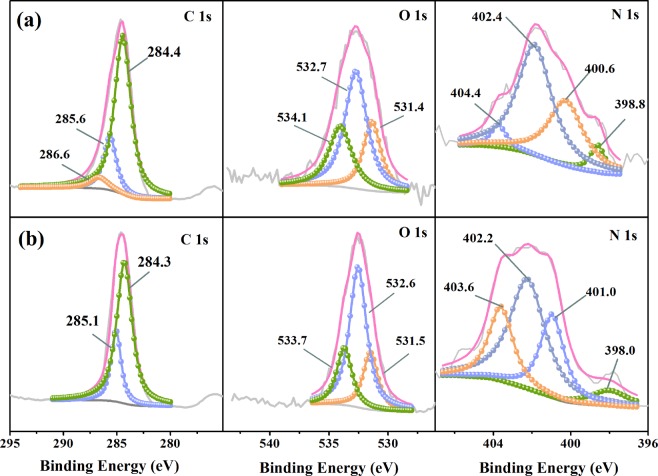
High-resolution XPS spectra for C 1 s, O 1 s and N 1 s of the mesoporous carbon before (**a**) and after (**b**) adsorption of AR88.

The high-resolution XPS spectra for C 1s, O 1s and N 1s of the samples are presented in Fig. 6 and the relative contents of each functional group calculated from their peak areas are summarized in Table 4. The peaks of C 1s spectra with binding energy (BE) at 286~ 287, 285~ 286 and ~ 284.6 eV were associated with the C=O, C-O and graphitic C-C, respectively^36^. Four evident peaks could be detected in N 1 s spectra, corresponding with the pyridinic N (N-6, ~399 eV), pyrrolic N (N-5, ~400 eV), quaternary-N (N-Q, 401~ 403 eV) and oxidized-N (N-X, 403.5 and 406 eV), respectively^37^.

**Table 4:** Relative contents (%) of each element and functional groups of the sample pre-oxidized at 200 °C before and after adsorption.

Binding Energy (eV)	Before	After
C 1s	88.30	81.97
C-C (~ 284.6)	74.71	75.34
C-O (285–286)	19.54	24.66
C=O (286–287)	5.75	–
O 1s	9.50	16.34
O=C (~ 531.5)	23.55	20.67
O-C (~ 532.6)	49.93	58.27
COOH (~ 533.7)	26.52	21.06
N 1s	2.21	1.69
N-6	5.77	4.29
N-5	33.69	27.11
N-Q	55.54	47.06
N-X	5.00	21.54

As shown in Fig. 6 and Table 4, after adsorption, the relative content of C=O declined significantly from 5.75 to 0.00%, revealing that it might be evolved in the adsorption process. It was reported that C=O was a kind of Lewis basic group, which had a strong attraction for acid molecules by electrostatic interaction. After adsorption, the contents of N-6, N-5 and N-Q became lower than those before adsorption, while that of N-X increased significantly from 5.00% to 21.54%. This indicates that N-6, N-5 and N-Q could participate in the reaction and converted to N-X during the adsorption process. This phenomenon might be related to the occupation of basic functional groups (pyridine-N) during the adsorption process, which could attract phenolic hydroxyl and sulfonic group in AR88 by electrostatic interaction^38^. Normally, pyridine-N is bonded to two neighbouring C atoms with the location along with the edges of the carbon planes, which are considered as Lewis bases and readily interact with the acidic AR88 molecules^39^. According to Gong’s work^40^, the nitrogen atom in pyridinic C-N group can form the strong hydrogen bonding interaction with dye molecule. The porous carbon was also detected using Raman analysis and related spectra were plotted in Fig. S3. The characteristic peaks located at approximately and cm can be assigned to G band (the in-phase vibration of the sample lattice) and D band (the disorder band)^41^. In general, a smaller D/G ratio of peak intensity reveals a perfect graphitic structure. The D/G ratio was found to be 1.68 revealing a graphitic structure with disordered carbons was created in the mesoporous carbon after high-temperature carbonization, which could facilitate the formation of π-π dispersion interaction between graphic C with the aromatic rings in AR88, leading to enhanced adsorption^42^.

#### The relationship between adsorption capacity and textural properties

Fig. S2(d) shows the N_2_ adsorption-desorption isotherm of the sample (with the weight ratio of 1:3, carbonized at 900 °C and pre-oxidized at 200 °C) before and after adsorption of AR88. Compared with the fresh one, the sample after adsorption presented evidently a lower nitrogen adsorption capacity during all relative pressure range. Its textural properties after adsorption are listed in Table 1. It can be seen that its S_BET_, V_tot_, V_meso_ and V_mic_ were dramatically decreased compared with the sample before adsorption. As shown in Fig. 3(d), the sample after adsorption possessed similar mesopore size distribution as the fresh one, while the peak area was clearly lower. The results suggest that the mesopores of the carbon were occupied mostly by the AR88 molecules, resulting in the lower porosity of the carbon after adsorption.

In addition, the relationships between the AR88 adsorption capacities and the textural properties of prepared samples were investigated (Fig. S4), and their correlation coefficient (R^2^) are listed in Table 5. It can be found that the overall R^2^ of the adsorption capacity vs S_BET_, V_tot_ and V_meso_ was evidently higher than those of V_mic_ for all of the prepared carbons. This indicates that they played dominated roles in the adsorption of AR88, while V_mic_ had a negligible effect. AR88 was a kind of macromolecules with the molecular diameter at about 1.68 nm. As reported in previous studies, the pores with the diameter 2 to 3 times larger than the adsorbate molecular diameter could allow the adsorbate diffusion easily^43^. The AR88 with macromolecules could not be allowed to enter into narrow pores of carbon (<2 nm). Thus, the abundant mesoporous structure of prepared carbon in this study was preferred for the adsorption of AR88. The AR88 adsorption capacities were dramatically affected by the textural properties of prepared mesoporous carbon. The S_BET_ of the samples from different pre-oxidation conditions had a relatively lower correlation with adsorption capacities (0.874), compared with those prepared at different weight ratios (0.982) and carbonization temperature (0.978). This demonstrates that there might be other factors affecting the AR88 adsorption, except for the porous structure of carbon. For a better understanding of the factors responsible for the adsorption performance beyond porous structure, the AR88 adsorption capacities of the carbon with different pre-oxidization temperatures were normalized by S_BET_ (q/S_BET_), and the results are listed in Table 3. The sample without pre-oxidization presented the highest q/S_BET_ at 0.702, while those with pre-oxidization had lower q/S_BET_ values. The sample pre-oxidized at 240 °C exhibited the lowest value, i.e., only 0.596. This result presented a similar order with the N contents in each carbon (Table 3), suggesting that higher contents of N on the carbon surface might help its AR88 adsorption.

**Table 5:** Correlation (R^2^) between AR88 adsorption capacities and textural properties of prepared samples.

Preparation condition	Textural properties			
	S_BET_	V_tot_	V_mic_	V_meso_
Weight ratio	0.982	0.942	0.568	0.947
Carbonization temperature	0.978	0.996	0.884	0.997
Pre-oxidation temperature	0.874	0.954	0.073	0.957
Overall	0.895	0.840	0.191	0.819

**Figure 7:**
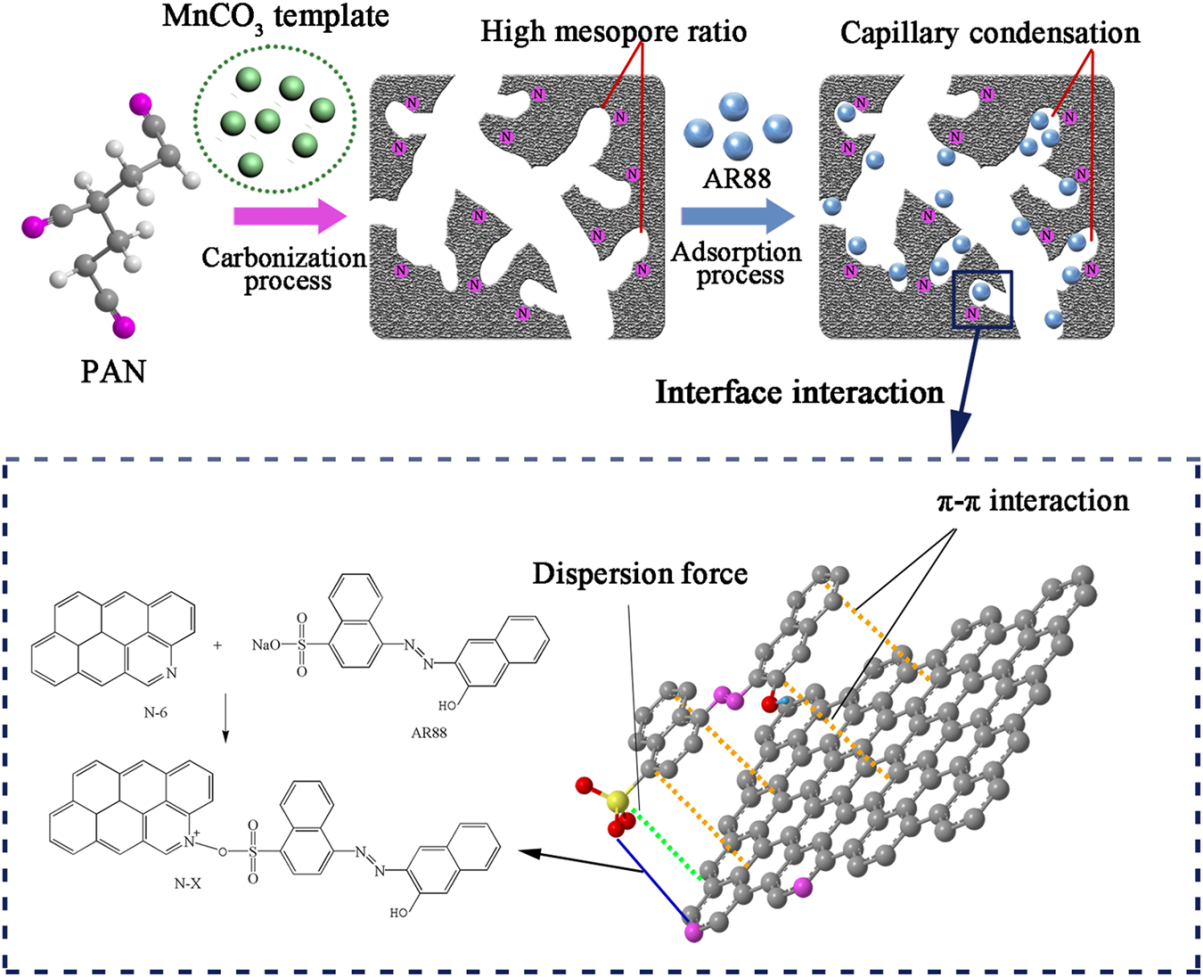
Schematic illustration showing the AR88 adsorption mechanism by mesoporous carbon: the capillary condensation effect of mesopores and the interactions between porous carbon and AR88.

Combined with the results of FTIR and XPS, it can be assumed that both physical and chemical interaction could happen between the AR88 and prepared carbon. Considering these results, the possible adsorption mechanisms of AR88 molecules by mesoporous carbon can be proposed in Fig. 7. First, the rich mesopores in porous carbon enhance the capillary condensation, which facilitates the diffusion and adsorption of AR88 molecules. Besides, the AR88 adsorption could also be affected by the electrostatic interaction between acid AR88 molecules and basic groups on carbon surface (including C=O and pyridine-N), and π-π dispersion interaction between aromatic rings of AR88 and carbon basal plane with the presence of N atoms.

#### Adsorption isotherm

**Figure 8:**
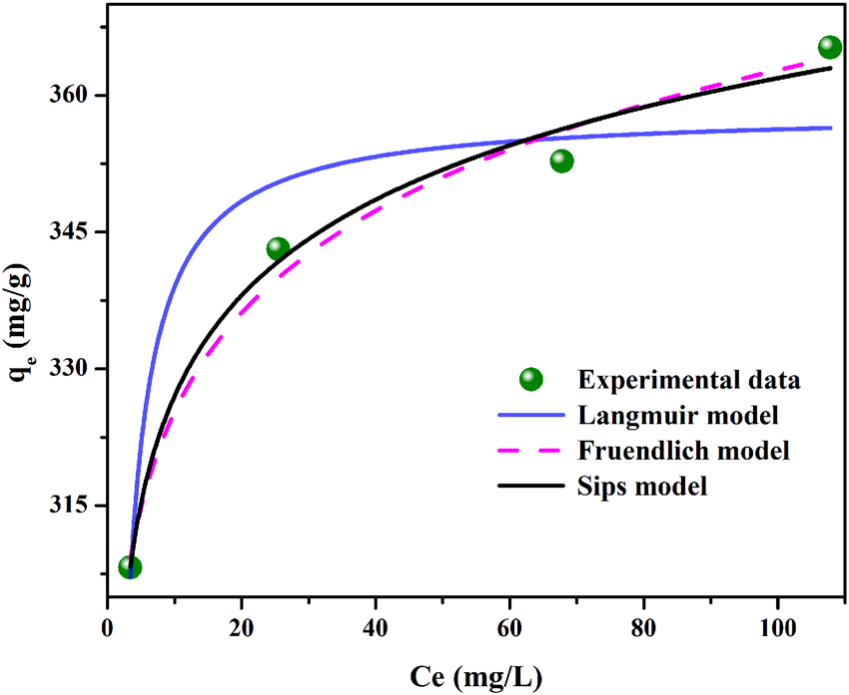
Non-linear fitting of Langmuir, Freundlich and Sips model for AR88 adsorption by mesoporous carbon.

To better understand the AR88 adsorption process by the prepared mesoporous carbon, the correlation between adsorption equilibrium concentration and the adsorption capacity for AR88 was evaluated at 25 °C. From the adsorption isotherm curve plotted in Fig. 8, the AR88 adsorption amount was enhanced gradually with increasing the equilibrium concentration and reached a plateau at a relatively high equilibrium value.

Adsorption isotherm could help understand the interaction between adsorbents and adsorbates, which is essential for the optimal utilization of adsorbents. Three isotherm models, i.e. Langmuir, Freundlich and Sips model, were applied to investigate the interaction of mesoporous carbon with AR88. The non-linear fitting of the three models is plotted in Fig. 8 and their parameters, together with R^2^, are given in Table 6. The R^2^ of each model was in the order of Sips (0.989) > Freundlich (0.987) > Langmuir (0.922), demonstrating that Sips and Freundlich could provide a better description of the process of AR88 adsorption on the prepared mesoporous carbon. The isotherm curve presented a sustainable growth with the increase of C_e_ and q_m_ predicted by Sips model was 580.3 mg/g, indicating that more AR88 could be adsorbed at higher initial concentrations. Furthermore, the γ value was only 0.1, demonstrating that the adsorption process could not belong to monolayer sorption and some interactions might occur among adsorbed AR88 molecules.

**Table 6:** Results of isotherm and kinetic model fitting.

Model	Parameter	Value	R^2^
Isotherm			
Langmuir	q_m_ (mg/g)	358.3	0.922
	K_L_ (L/mg)	0.570	
Fruendlich	K_F_ ((mg/g)/(L/mg)^1/n^)	291.6	0.987
	1/n	0.047	
Sips	q_m_ (mg/g)	580.3	0.989
	K_S_ (mL/mg)	1.183	
	γ	0.100	
Kinetic			
Pseudo-1st-order	K_1_ (min^−1^)	0.092	0.989
	q_e_ (mg/g)	33.4	
Pseudo-2nd-order	K_2_ (g/(mg·min))	0.009	1.000
	q_e_ (mg/g)	312.5	
Intraparticle diffusion	K_p1_ (mg/g·min^0.5^)	7.29	0.994
	C_1_	274.5	
	K_p2_ (mg/g·min^0.5^)	2.89	0.964
	C_2_	292.2	
	K_p3_ (mg/g·min^0.5^)	0.18	0.753
	C_3_	309.7	

#### Kinetics study

**Figure 9:**
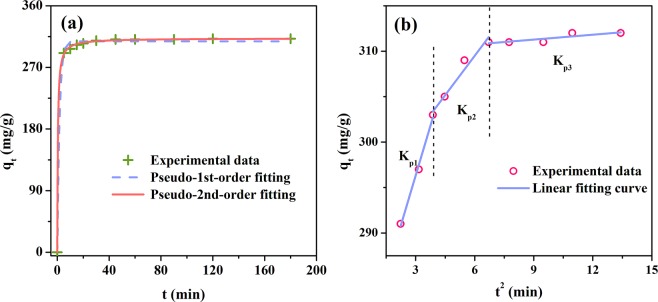
Non-linear fitting of pseudo-1^st^-order and pseudo-2^nd^-order (**a**) for AR88 adsorption by mesoporous carbon, and (**b**) linear fitting of the intraparticle diffusion model.

Figure 9 shows the AR88 adsorption capacity (q_t_) by prepared mesoporous carbon against time. It can be seen that the adsorption capacity increased dramatically to 290 mg/g within the initial 5 min, indicating that large quantities of AR88 molecule were rapidly uptake by prepared carbon. Subsequently, the increase of adsorption capacity was slow down and no more AR88 could be adsorbed after 45 min, suggesting that the adsorption equilibrium was approached.

The pseudo 1^st^ order, pseudo 2^nd^ order and intrparticle diffusion models were applied to simulate the adsorption process, whose fitting curves are presented in Fig. 9. It can be seen that the adsorption rate was high at the initial stage and gradually became lower after 5 min and the uptake of AR88 turned to be saturated after 30 min, revealing that the adsorption process finished rapidly. This might be due to the abundant mesoporous structure in carbon, which could decrease the diffusion resistance of AR88 molecules. The parameters for both pseudo-1^st^-order and pseudo-2^nd^-order are presented in Table 6. According to R^2^ value, the fitting dgree of pseudo-2^nd^-order model (R^2^ = 1.0) was higher than that of pseudo-1^st^-order (R^2^ = 0.989), indicating that the pseudo-2^nd^-order model could describe the adsorption process better. Pseudo-2^nd^-order model assumes that the adsorption rate is determined by the square of the vacant site number that has not been occupied by adsorbents and the adsorption process was controlled by the chemical effect. The desirable fitting of pseudo-2^nd^-order to experimental data suggested that the adsorption process could belong to chemisorption and the adsorption involves the electron transfer or/and sharing. The results are in a good agreement with the variation of surface chemistry before and after adsorption (Figs. 5 and 6).

The linear fitting of the intraparticle diffusion model to experimental data is depicted in Fig. 9(b), in which three linear stages could be observed, with the slopes as K_p1_, K_p2_ and K_p3_, respectively. The parameters, including the slopes, intercept and R^2^ values, are listed in Table 6. It could be seen that the slope of the three stages was in the order of K_p1_ > K_p2_ > K_p3_, indicating that the equilibrium was gradually obtained. The first and sharp liner portion of plots reveals that the boundary layer diffusion limits the AR88 adsorption. The last two gentle stages indicate that the occurrence of intraparticle diffusion is the adsorption limiting step^44^. In addition, all C values were relatively high and far from the origin, demonstrating that the intraparticle diffusion was not the sole rate-controlling step, which was ascribed to the rich mesoporous structure in prepared carbon that could effectively decrease the diffusion resistance of AR88.

In summary, the nitrogen-containing graphitic mesoporous carbon could be successfully prepared by using PAN as both carbon and nitrogen resources and MnCO_3_ as template and catalyst precursors. The graphitized degree of prepared carbon could be evidently elevated via the introduction of MnCO_3_. The weight ratios of carbon and template precursor, carbonization temperature and pre-oxidation temperature played vital roles for the textural properties of prepared mesoporous carbon, which affect significantly their AR88 adsorption behavior. Moreover, the carbon- and nitrogen-containing groups on the surface of mesoporous carbon also had an influence on the adsorption capacity of AR88. The rich graphic carbon tended to form the π-π dispersion interaction with the aromatic rings in AR88. which contributed to the adsorption process. The π-π dispersion interaction and electrostatic interaction Sips model could give a better description for the AR88 adsorption by the prepared mesoporous carbon, with the γ value at 0.01, suggesting that the adsorbent surface might not be homogenous or/and there might be some interactions among adsorbates. Furthermore, the pseudo 2^nd^-order model fitted the experimental data better than the pseudo 1^st^-order model, confirming the chemisorption of AR88. The intraparticle diffusion model certified that the intraparticle diffusion was not the rate-determining step of adsorption, which was attributed to the abundant mesoporous structure in the prepared mesoporous carbon.

## Methods

### Materials

PAN, MnCO_3_, H_2_SO_4_ were provided from Kelong chemical reagent factory, Chengdu, China. AR88 was purchased from Aladdin Chemistry Co., Ltd. All reagents were used in this study without further treatment.

### Preparation of mesoporous carbon

**Figure 10:**
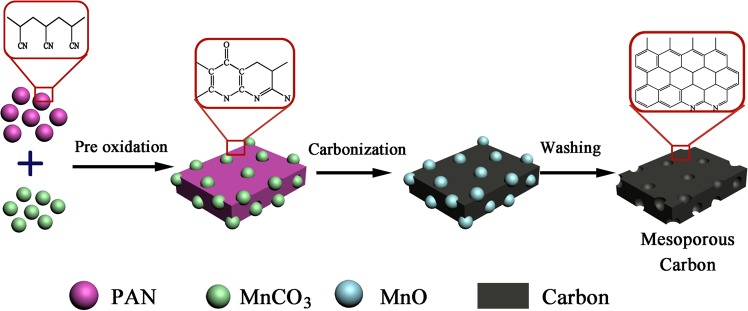
Schematic illustration showing the preparation route of PAN-mesoporous carbon.

The received PAN and MnCO_3_ with particle size below 200 mesh were adequately mixed at specified weight ratios at 2:1, 1:1, 1:2, 1:3, 1:4 and 1:5, respectively. The mixtures were firstly pre-oxidized at 200 °C for 60 min in air and subsequently heated up to 900 °C with a heating rate of 5 °C/min under a nitrogen atmosphere using a horizontal tube furnace. After 1 h of carbonization, the samples were cooled down to room temperature, followed by acid washing using 1.8 mol/L of H_2_SO_4_ solution for several times until the concentrations of Mn^2+^ lower than 0.01 mg/L. Finally, samples were washed with distilled water to neutral and dried at 105 °C overnight. The related preparation route is illustrated in Fig. 10. Furthermore, the effects of the carbonization temperature (700, 800, 900 and 950 °C) and pre-oxidation (180, 200 and 240 °C) on the prepared mesoporous carbon were also evaluated.

### Characterization of mesoporous carbon

Thermogravimetric method was adopted to simulate the carbonization process of PAN. The received PAN, MnCO_3_ powder and their mixture with and without pre-oxidation at 200 °C for 1 h were transferred into a TG analyzer (Ta-50, Shimadzu, Japan) and heated to 1000 °C under N_2_ atmosphere with a heating rate of 5 °C/min. The transformation of Mn and the graphitization degree of prepared carbon were performed on an X-ray diffractometry (X-Pert PRO MPD, PANalytical B.V., Netherland), whose patterns were recorded with a resolution of 0.026 in the 2θ ranging from 10° to 80°. And the interplanar spacing (d) of graphite carbon was calculated from Bragg’s law. The porous structure of prepared carbons was characterized by using high-resolution transmission electron microscope (HRTEM) (Libra 200 FE, ZEISS, Germany). N_2_ adsorption-desorption method was applied for analyzing the porous structure of prepared mesoporous carbon using a surface area analyzer (ASAP2460, Micromeritics, USA). Before measurement, all carbons were degassed at 250 °C for 8 h to remove volatile impurities. The BET surface area was determined using the Brunauer-Emmett-Teller (BET) equation in the relative pressure ranging from 0.05 to 0.35. The microporous volume (V_mic_) and total porous volume (V_tot_) were calculated from the t-plot method, while the V_meso_ and the mesopore size distribution were obtained from the BJH model. The surface chemistry of mesoporous carbon was characterized by Fourier transform infrared (FITR) spectroscopy (Nicolet 6700, Thermo Scientific, USA), X-ray photoelectron spectroscopy (XSAM800, KRATOS, UK) and Raman spectroscopy (HORIBA Jobin Yvon). For FTIR analysis, the dried carbon was mixed with KBr powder adequately and then pressed into a disk with a thickness of 0.8 mm. The FTIR spectra were recorded with a step size of 2 cm^−1^ in the scanning from 4000 to 400 cm^−1^. XPS analysis was performed at a pressure of 10^−7^ Pa using a monochromatic Al Kα X-ray source. The XPS Peaks software was used for data analyzing and all spectra curves were peak fitted with Gaussian-Lorentzian convoluted functions and Shirley’s background. Raman tests were carried out using Labram HR spectrometer at laser excitation of 532 nm and the power of 1 mW.

### AR88 adsorption experiments

Prepared carbons with different template contents, carbonization temperature and pre-oxidation temperature were adopted as the adsorbents for the removal of AR88, which was performed using 500 mL of Erlenmeyer flasks at 25 °C. 250 mL of AR88 solutions (250 mg/L) and 0.2 g of carbon were added into the flasks and then they were agitated at 180 rpm in a thermostatic shaker for 30 min. The adsorption capacities of the adsorbents were obtained from the absorbance of AR88 solutions before and after adsorption at a wavelength of 503 nm. The adsorption capacity and removal efficiency were calculated from Eqs. (1) and (2), respectively:

1 $${\rm{q}}=\frac{({{\rm{C}}}_{0}{-C}_{{\rm{e}}})\times V}{{\rm{W}}}$$

2 $${\rm{\eta }}=\frac{{{\rm{C}}}_{0}{-C}_{{\rm{e}}}}{{{\rm{C}}}_{0}}\times 100 \% $$

where q (mg/g) is the adsorption capacity, C_0_ is the initial concentration of AR88, C_e_ is the equilibrium concentration (mg/g), V is the volume of the AR88 solution, W (g) is the weight of adsorbent, and η (%) is the removal efficiency. All experiments were carried out in triplicate at the same time.

#### Adsorption isotherm

Three adsorption isotherms, including Langmuir, Freundlich and Sips model, were applied to explain the relationship between adsorbent and adsorbate and provide basic data to evaluate the adsorption process on practical application. Five different initial concentrations (150, 200, 250, 300 and 400 mg/L) were adopted for isotherm analysis. Langmuir model was an empirical equation summarized from large quantities of experiments, which was widely investigated in wastewater adsorption treatment. Four assumptions were proposed for Langmuir model: First, adsorbates can only be adsorbed on the surface of adsorbents in monomolecular type; Second, the effects of the surface are even and each adsorption center have same energy; Third, no interaction exists among adsorbed molecules; Fourth, the dynamic balance of adsorption-desorption can be realized at some condition. The Langmuir equation is given in Eq. (3):

3 $${{\rm{q}}}_{{\rm{e}}}=\frac{{{\rm{q}}}_{{\rm{m}}}{{\rm{K}}}_{{\rm{L}}}{{\rm{C}}}_{{\rm{e}}}}{{1+K}_{{\rm{L}}}{{\rm{C}}}_{{\rm{e}}}}$$

where q_e_ is the equilibrium adsorption capacity, C_e_ (mg/L) is the equilibrium concentration, q_m_ (mg/g) is the theoretical maximum adsorption capacity, and b (mg/L) is the Langmuir constant related to the free energy of adsorption^45^. A higher b value confirms the high interaction stability between adsorbents and adsorbates. Freundlich model takes the heterogeneity of the adsorbent surface into account. It assumes that the adsorbent surface is not homogeneous and the adsorption is in multi-layer type. Moreover, several interactions can exist among adsorbate molecules. The Freundlich equation is presented in Eq. (4):

4 $${{\rm{q}}}_{{\rm{e}}}{=K}_{{\rm{f}}}{{\rm{C}}}_{{\rm{e}}}^{1/n}$$

where K_f_ ((mg/g)/(L/mg)^1/n^) is the constant related to the adsorption capacity and n represents the process intensity.

Sips model is the modified Langmuir model, whose equation is in Eq. (5):

5 $${{\rm{q}}}_{{\rm{e}}}{=q}_{{\rm{m}}}\frac{{{(K}_{{\rm{S}}}{{\rm{C}}}_{{\rm{e}}})}^{{\rm{\gamma }}}}{{1+(K}_{{\rm{S}}}{{\rm{C}}}_{{\rm{e}}}{)}^{{\rm{\gamma }}}}$$

where K_S_ (mL/mg) is the Sips isotherm constant and γ (dimensionless) is the Sips exponent^46^. For Sips model, a parameter γ was proposed to identify the homogeneity of adsorbent surface, thus it could be regarded as the combination of Langmuir model and Freundlich model. Once the value of γ is 1, the equation is equal to Langmuir model, proving the surface of adsorbent was homogenous^47^.

#### Kinetics study

Adsorption kinetics could help understand the adsorption mechanism and rate of mesoporous carbon, which is an important method to investigate the mass transfer process of adsorption. 0.2 g mesoporous carbon was added into 250 mL of 250 mg/L AR88 solution and the adsorption capacity was measured every five minutes. Three mathematical models, including pseudo-first-order, pseudo-second-order and intraparticle diffusion equation, were used to describe the experimental data of adsorption kinetics. The pseudo-first-order model is represented in Eq. (6):

6 $$\mathrm{ln}({{\rm{q}}}_{{\rm{e}}}{-q}_{{\rm{t}}}){=\text{lnq}}_{{\rm{e}}}{-K}_{1}{\rm{t}}$$

where q_e_ (mg/g) and q_t_ (mg/g) are the amounts of AR88 adsorbed at equilibrium and time t (min), respectively, while K_1_ (min^−1^) is the rate constant. The K_1_ value was obtained from the slope of ln(q_e_-q_t_) vs t.

The pseudo-second-order model was expressed by the Eq. (7) presented as follows:

7 $$\frac{{\rm{t}}}{{{\rm{q}}}_{{\rm{t}}}}=\frac{1}{{{\rm{K}}}_{2}{{\rm{q}}}_{{\rm{e}}}^{2}}+\frac{{\rm{t}}}{{{\rm{q}}}_{{\rm{e}}}}$$

where K_2_ (g/(mg·min)) is the rate constant pseudo-second-order kinetic model, which was calculated from the slope of the linear plots t/q_t_ against t.

The intraparticle diffusion models could describe the internal particle diffusion effect, whose equation was presented as Eq. (8):

8 $${{\rm{q}}}_{{\rm{t}}}{=K}_{{\rm{p}}}{{\rm{t}}}^{0.5}+C$$

where K_p_ (mg/(g·min)) is the intra-particle diffusion rate constant. K_p_ and C were the slope and intercept of the linear plots of q_t_ to t^0.5^, respectively.

## Supplementary information

Surpporting Imformation.
