# Supplementary material for: Preparation of a novel nitrogen-containing graphitic mesoporous carbon for the removal of acid red 88
Source: Sci Rep. 2020 Jan 28;10:1353. doi: 10.1038/s41598-020-57823-z (PMC6987219; doi:10.1038/s41598-020-57823-z)
Supplement: Supplementary file 1 — Surpporting Imformation. [file 41598_2020_57823_MOESM1_ESM.docx]

**Supporting information**

**Preparation of a novel nitrogen-containing graphitic mesoporous carbon for the removal of acid red 88**

**Qiying Zhou^a, b+^, Wenhua Chen^a, b+^, Xia Jiang^a, b^*, Hongying Liu^a, b^, Shenggui Ma^a, b^, Bangda Wang^a, b^**

^a^ College of Architecture and Environment, Sichuan University, Chengdu 610065, China

^b^ National Engineering Research Centre for Flue Gas Desulfurization, Chengdu 610065, China

* Corresponding author. E-mail: xjiang@scu.edu.cn (X Jiang)

**^+^** These authors contributed equally to this work





**Figure S1. TG and DTG curves of PAN/MnCO3 mixture (the weight ratio is 1:3) with and without** **pre-oxidation.**





**Figure S2.** **N2 adsorption-desorption isotherms at 77 K of the samples prepared from different conditions (weight ratio of PAN and MnO (a), carbonization temperature (b), pre-oxidation temperature (c), and after adsorption (d)).**





**Figure S3. Raman spectra of PAN and porous carbon prepared (the weight ratio of 1:3, pre-oxidized at 200 °C and carbonized at 900 °C)**





**Figure S****4. Linear relationship between the AR88 adsorption capacities and the textural properties of prepared samples (S_BET_ (a), V_mic_ (b), V_tot_ (c) and V_meso_ (d). WR: Weight ratio of PAN and MnO, CT: Carbonization temperature (°C), PT: Pre-oxidation temperature (°C). Symbols represented the experimental data while lines represented the linear fitting curve)**
